# Attitudes of Older Adults With Chronic Musculoskeletal Pain Toward Virtual Reality

**DOI:** 10.1093/geroni/igaa057.655

**Published:** 2020-12-16

**Authors:** Lynn Nakad, Barbara Rakel

**Affiliations:** University of Iowa, Iowa City, Iowa, United States

## Abstract

Older adults are especially susceptible to chronic musculoskeletal (MSK) pain. Pharmacological treatment often provides inadequate relief and overuse can lead to adverse events. Preliminary research has demonstrated the effectiveness of virtual reality (VR) distraction therapy for chronic pain. Understanding attitudes of older adults towards VR distraction therapy will help develop and optimize this therapy for this population. Therefore, the purpose of this study is to explore the attitudes and treatment acceptability towards the use of immersive VR distraction therapy of older adults suffering from chronic MSK pain. This descriptive, exploratory study used mixed methods. Data collection consisted of a consent process, eligibility screening, survey completion, 2 VR simulations (passive and active) lasting 10-minutes each, and either a focus group or interview. Survey data was used to measure: pain intensity and interference, pain catastrophizing, treatment acceptability, usability, and side effects. A total of 21 older adults completed the study. Treatment acceptability was high with an average score of 32.5 out of 40. However, average usability scores (62.9 out of 100) indicated a need for system improvements. Few participants (14%) experienced moderate to severe side effects. The following themes were identified: 1) VR is an enjoyable distraction; 2) Perceived effectiveness depends on chronic pain experience; 3) VR simulation experiences should be individualized; 4) Design considerations to improve usability in older adults; 5) Recommendations for future directions. The findings from this study can inform intervention design considerations and future directions for the use of VR technology for chronic pain management in older adults.

